# Supplementary material for: Uncovering developmental time and tempo using deep learning
Source: Nat Methods. 2023 Nov 23;20(12):2000–10. doi: 10.1038/s41592-023-02083-8 (PMC10703695; doi:10.1038/s41592-023-02083-8)
Supplement: Supplementary file 1 — Supplementary Notes 1 and 2, Figs. 1–4, Tables 1 and 2 and References. [file 41592_2023_2083_MOESM1_ESM.pdf]

nature methods

Article

https://doi.org/10.1038/s41592-023-02083-8

Uncovering developmental time and tempo using deep learning

In the format provided by the authors and unedited

Table of Contents

Supplementary Notes 1-2

Supplementary Figures 1-4

Supplementary Tables 1-2

Supplementary References

Supplementary Note 1

Overview of early zebrafish development

Developmental stages and the corresponding transitions between them have been particularly well described for zebrafish embryos, since large numbers of externally laid eggs can be obtained and the formation of the body plan is easily observable with single-cell resolution due to the transparent nature of the embryos[1,2]. In 1995, Chuck Kimmel published his influential atlas of zebrafish development, in which he described a set of characteristic features that can be observed during the first hours of embryogenesis under standardized laboratory conditions[3]. In zebrafish, the cleavage period is characterized by rapid and constant synchronous meroblastic divisions. In the blastula period, the embryo transitions from maternally provided transcripts to initiate the transcription of its own genome[4]. From this point on, the cell cycle lengthens and cell divisions occur asynchronously under nucleocytoplasmic control during embryogenesis[5]. At the end of the blastula period, large-scale cell movements begin and continue throughout the gastrula stage. During the gastrula period, the germ layers are formed, which provide the organization of the future inner-to-outer body axis. This takes place under the influence of signaling molecules, among which BMP, Nodal, RA, FGF and Wnt participate in shaping the anterior-posterior and dorsal-ventral axes[6-10]. The subsequent stage is characterized by the formation of somites – segmentally articulated parts of the mesoderm –, organogenesis and tail development.

Supplementary Note 2

Benchmarking of the Twin Network against other techniques

A previous technique based on vector diffusion maps[11] described developing processes by automatically ordering a sequence of images without *a priori* knowledge[12]. Benchmarking against the present model (Supplementary Fig. 2) revealed that our model performs at least on par with the reference method in the task of image ordering (Supplementary Fig. 3). Our model shows comparable deviation from the expected ground truth (Supplementary Fig. 3a-g), while the absolute deviation from ground truth is less than the prediction of the reference model. This trend is emphasized when temporal resolution is reduced stepwise (Supplementary Fig. 3h). Comparing the absolute deviation between both models, the Twin Network offers increased precision (range of p-values from 0.016 to 0.078) in image ordering compared to the vector diffusion map-based approach.

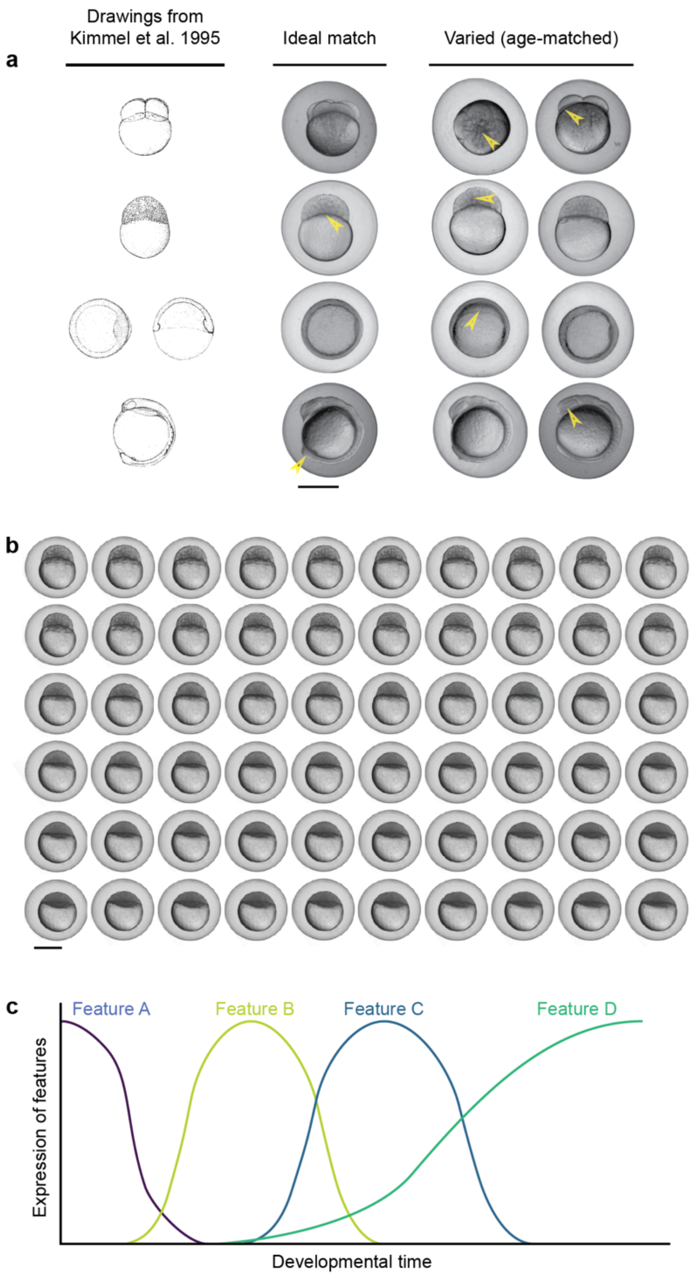

Supplementary Figure 1 | Stages of zebrafish development. (a) Variation of embryonic phenotypes compared to the idealized drawn stages in the classical atlas by Kimmel et al. 1995[3]. Yellow arrowheads point to example locations where phenotypic features differ from the idealized depictions in the staging atlas. **(b)** Transitions between embryonic stages are fluid, and finding sharp boundaries between embryonic stages can be difficult. A representative time series of a test embryo is shown. The first image on the top left shows the embryo at 2.5 hpf. The following images were taken with average acquisition intervals of 134.8 s and are shown in rows from left to right. The last image in the time series at the bottom right was taken at 4.75 hpf. **(c)** Different phenotypic features appear at different stages of embryogenesis, combinations of which are often employed to determine embryonic stages. Scale bars: 500 µm.

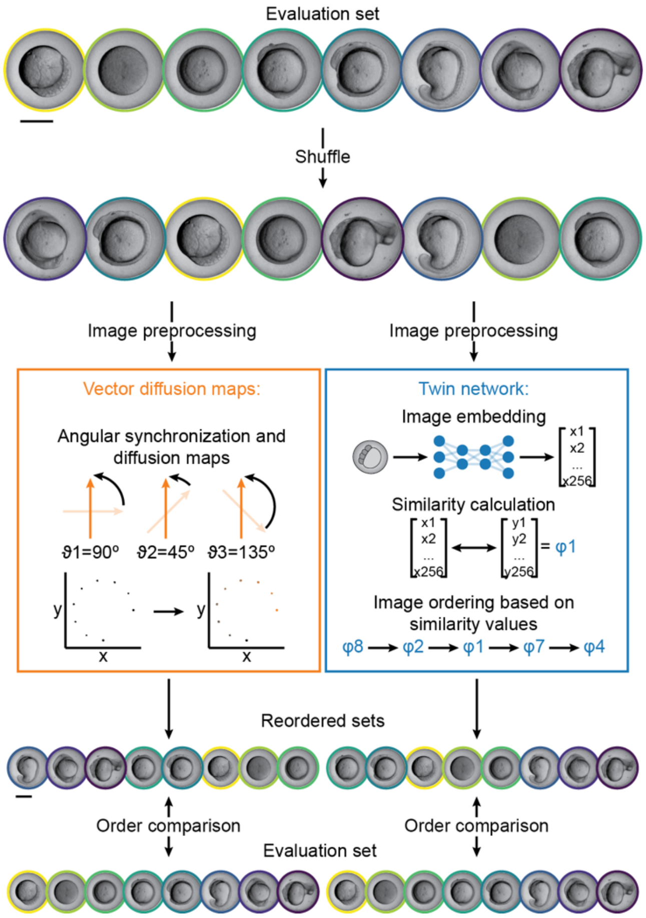

Supplementary Figure 2 | Strategy to benchmark the Twin Network in comparison to vector diffusion map techniques. A shuffled data set of embryo images with known original order was used to evaluate temporal ordering of image sets by vector diffusion maps as described by Dsilva et al. 2015[12] and by our Twin Network. For the evaluation of vector diffusion maps, the software by Dsilva et al.[12] was used to predict the image order. For our Twin Network, embeddings were generated for all images of the data set, and similarities were calculated between all embeddings. The image order was determined by iteration over the similarity values and selection of subsequent images based on the highest similarity values in the data set. For both the vector diffusion map and Twin Network model, the predicted order was compared to the true image order. This process was repeated for a decreasing number of images per data set and increasing time intervals between acquisition time points. Results are shown in Supplementary Fig. 3. Scale bars: 500 µm.

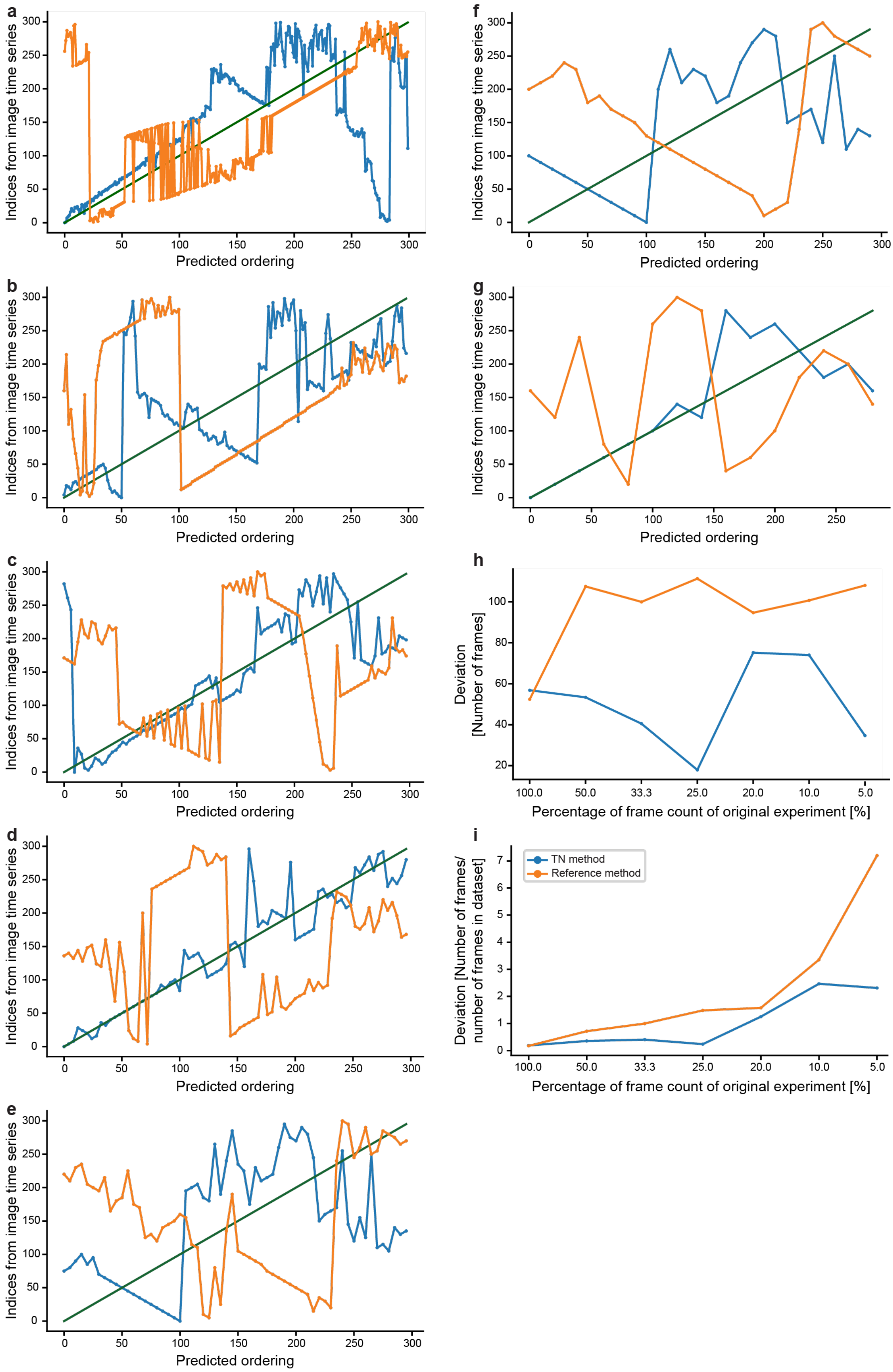

Supplementary Figure 3 | Benchmarking the Twin Network against vector diffusion maps. (a-g) Predicted image order indices compared to true indices, as predicted by the Twin Network (blue) and the reference method (orange). The underlying ground truth value is plotted in green. The analyses were performed on the same data set with increasing step sizes: From

the data set, every 2nd image was used in (b), every 3rd was used in (c), every 4th in (d), every 5th in (e), every 10th in (f) and every 20th in (g). **(h)** Absolute average deviation for different shares of images in the data set for both the Twin Network method (blue) and vector diffusion maps (orange). On average, the Twin Network method shows a lower absolute deviation of the predicted order compared to the true indices. **(i)** Data set-size adjusted deviation is shown for the test embryo data set with 100%, 50%, 33%, 25%, 10% and 5% of the original images. The absolute deviation shown in (h) is divided by the number of images used in the data set, corresponding to the step sizes described for (a-g). With fewer images in the data set, the Twin Network method shows increased performance in comparison to the reference method. Analysis of one embryo is shown, representative for n = 2 analyzed embryos.

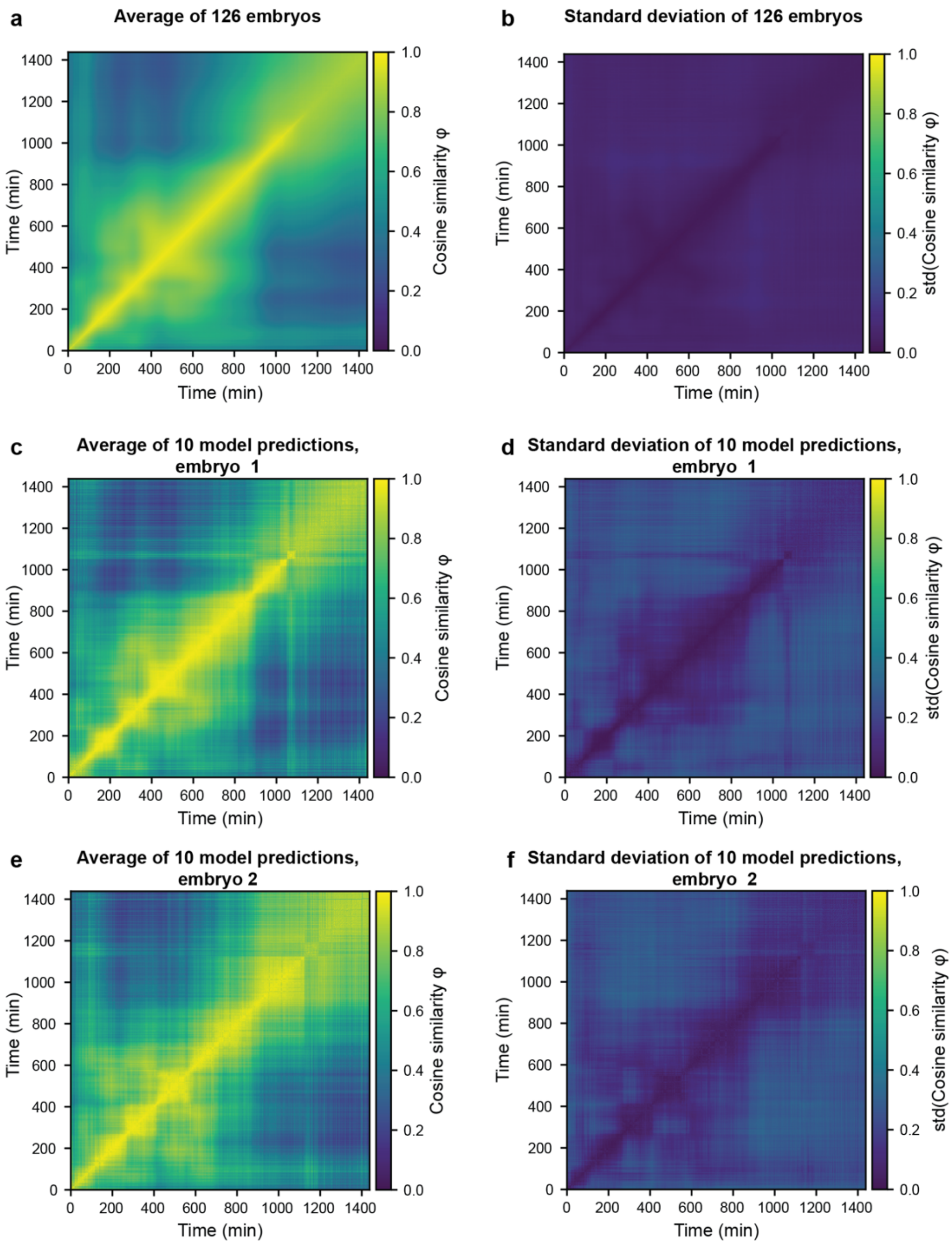

Supplementary Figure 4 | Assessing technical and biological variability for autostaging. Self-similarity matrices calculated using 10 independently trained models of the Twin Network to evaluate technical and biological variability. For each zebrafish embryo, a self-similarity matrix was calculated using each of the 10 trained models, and the element-wise average was calculated. **(a,b)** Mean and standard deviations matrices of the self-similarity matrices over n = 126 test embryos. **(c,e)** Average self-similarity matrices of 10 model predictions for two representative embryos. **(d,f)** Standard deviation of the self-similarity matrices of 10 model predictions for two representative embryos.

Supplementary Table 1. Overview of genetic backgrounds and acquisition parameters for zebrafish embryo image time series using high-content microscopy.

Data set	Cross of zebrafish lines	Brightfield illumination	LED module GFP (470 nm, 400 mW)
1	*Tg(sebox:EGFP)* × *TE*	100%, 100 ms	10%, 100 ms
2	*Tg(sebox:EGFP)* × $sqt^{+/-}$	100%, 100 ms	2%, 100 ms
3	*Tg(sebox:EGFP)* × *Tg(gsc:TurboRFP)*	100%, 100 ms	2%, 150 ms
4	*Tg(sebox:EGFP)* × $sqt^{+/-}$,<br>*Tg(sebox:EGFP)* × *Tg(gsc:TurboRFP)*,<br>$sqt^{+/-}$ × $sqt^{+/-}$	100%, 100 ms	2%, 150 ms
5	*Tg(sebox:EGFP)* × *TE*	100%, 100 ms	2%, 150 ms
6	*Tg(sebox:EGFP)* × *Tg(gsc:TurboRFP)*	100%, 100 ms	2%, 150 ms
7	*Tg(sebox:EGFP)* × $sqt^{+/-}$	100%, 100 ms	2%, 150 ms
8	*Tg(sebox:EGFP)* × $sqt^{+/-}$,<br>*Tg(sebox:EGFP)* × *TE*	100%, 100 ms	2%, 150 ms
9	*Tg(sebox:EGFP)* × *Tg(sebox:EGFP)*	100%, 100 ms	2%, 150 ms
10	*Tg(sebox:EGFP)* × *Tg(sebox:EGFP)*	100%, 100 ms	2%, 150 ms
11	*Tg(sebox:EGFP)* × *Tg(sebox:EGFP)*,<br>*Tg(sebox:EGFP)* × *Tg(lhx1a:EGFP)*,<br>*Tg(sebox:EGFP)* × *Tg(gsc:GFP)*,<br>*Tg(gsc:GFP)* × *Tg(gsc:GFP)*	100%, 100 ms	2%, 150 ms
12	*Tg(sebox:EGFP)* × *Tg(sebox:EGFP)*	100%, 100 ms	2%, 200 ms
13	*TE* × *TE*	100%, 100 ms	3%, 200 ms
14	*Tg(sebox:EGFP)* × *Tg(sebox:EGFP)*	100%, 100 ms	3%, 200 ms
15	*Tg(sebox:EGFP)* × *Tg(sebox:EGFP)*,<br>*Tg(sebox:EGFP)* × *TE*	100%, 100 ms	3%, 200 ms
16	*Tg(sebox:EGFP)* × *Tg(sebox:EGFP)*	100%, 100 ms	3%, 200 ms
17	*TE* × *TE*	100%, 100 ms	-
18	*TE* × *TE*	100%, 100 ms	-
19	*Tg(sebox:EGFP)* × *Tg(sebox:EGFP)*	100%, 100 ms	4%, 150 ms
20	*TE* × *TE*	100%, 100 ms	-
21	*Tg(sebox:EGFP)* × *Tg(sebox:EGFP)*	100%, 100 ms	5%, 100 ms
22	*Tg(sebox:EGFP)* × *Tg(sebox:EGFP)*	100%, 120 ms	5%, 100 ms
23	*Tg(sebox:EGFP)* × *Tg(sebox:EGFP)*	100%, 100 ms	4%, 100 ms
24	*Tg(sebox:EGFP)* × *Tg(sebox:EGFP)*	100%, 100 ms	5%, 100 ms
25	*Tg(sebox:EGFP)* × *Tg(sebox:EGFP)*	100%, 100 ms	4%, 110 ms
26	*Tg(sebox:EGFP)* × *Tg(sebox:EGFP)*	100%, 120 ms	4%, 100 ms
27	*Tg(sebox:EGFP)* × *Tg(sebox:EGFP)*	100%, 120 ms	4%, 100 ms
28	*Tg(sebox:EGFP)* × *Tg(sebox:EGFP)*	100%, 100 ms	5%, 100 ms
29	*Tg(sebox:EGFP)* × *Tg(sebox:EGFP)*	100%, 100 ms	5%, 100 ms
30	*Tg(sebox:EGFP)* × *Tg(sebox:EGFP)*	100%, 100 ms	5%, 100 ms
31	*Tg(sebox:EGFP)* × *TE*	100%, 100 ms	3%, 150 ms
32	*Tg(sebox:EGFP)* × *Tg(sebox:EGFP)*, *TE* × *TE*	100%, 100 ms	2%, 150 ms
33	*TE* × *TE*	100%, 100 ms	-
34	*Tg(sebox:EGFP)* × *Tg(sebox:EGFP)*	100%, 100 ms	2%, 150 ms

Supplementary Table 2. Overview of tested methods to apply the Twin Network for the analysis of developing processes.

Comparison samples 1			Comparison samples 2			Results	
Number of embryo samples	*Number of files per sample*	*Sample type*	*Number of embryo samples*	*Number of files per sample*	*Sample type*	*Number of comparison values*	*Plots*
1	1	Static embryo image	1	1	Static embryo image	1	Simple similarity score
1	1	Static embryo image	1	n	Embryo image time series	1 x n	n similarity scores → Similarity plot, autoregression at one time point
1	m	Embryo image time series	1	n	Embryo image time series	m x n	Predicted stages from m similarity plots → Course of development, autoregression at multiple time points
k	m	Embryo image time series	l	n	Embryo image time series	k x m x l x n	k x l courses of development → Distribution of developmental courses
k	1	Static embryo image	l	1	Static embryo image	k x l	k x l similarity scores → Variability of similarities

Supplementary References

1 Bailey, J., Oliveri, A. & Levin, E. D. Zebrafish model systems for developmental neurobehavioral toxicology. *Birth Defects Res C Embryo Today* **99**, 14-23 (2013). https://doi.org:10.1002/bdrc.21027

2 Marques, I. J., Lupi, E. & Mercader, N. Model systems for regeneration: zebrafish. *Development* **146**, dev167692 (2019). https://doi.org:10.1242/dev.167692

3 Kimmel, C. B., Ballard, W. W., Kimmel, S. R., Ullmann, B. & Schilling, T. F. Stages of embryonic development of the zebrafish. *Developmental Dynamics* **203**, 253-310 (1995). https://doi.org:https://doi.org/10.1002/aja.1002030302

4 Kane, D. A. & Kimmel, C. B. The zebrafish midblastula transition. *Development* **119**, 447-456 (1993). https://doi.org:10.1242/dev.119.2.447

5 Kane, D. A., Warga, R. M. & Kimmel, C. B. Mitotic domains in the early embryo of the zebrafish. *Nature* **360**, 735-737 (1992). https://doi.org:10.1038/360735a0

6 Schier, A. F. & Talbot, W. S. Nodal signaling and the zebrafish organizer. *Int J Dev Biol* **45**, 289-297 (2001).

7 Heisenberg, C. P. & Solnica-Krezel, L. Back and forth between cell fate specification and movement during vertebrate gastrulation. *Curr Opin Genet Dev* **18**, 311-316 (2008). https://doi.org:10.1016/j.gde.2008.07.011

8 De Robertis, E. M. & Kuroda, H. Dorsal-ventral patterning and neural induction in Xenopus embryos. *Annu Rev Cell Dev Biol* **20**, 285-308 (2004). https://doi.org:10.1146/annurev.cellbio.20.011403.154124

9 Heisenberg, C. P. *et al.* Silberblick/Wnt11 mediates convergent extension movements during zebrafish gastrulation. *Nature* **405**, 76-81 (2000). https://doi.org:10.1038/35011068

10 Schier, A. F. & Talbot, W. S. Molecular genetics of axis formation in zebrafish. *Annu Rev Genet* **39**, 561-613 (2005). https://doi.org:10.1146/annurev.genet.37.110801.143752

11 Singer, A. & Wu, H.-T. Vector diffusion maps and the connection Laplacian. *Communications on Pure and Applied Mathematics* **65**, 1067-1144 (2012). https://doi.org:https://doi.org/10.1002/cpa.21395

12 Dsilva, C. J. *et al.* Temporal ordering and registration of images in studies of developmental dynamics. *Development* **142**, 1717-1724 (2015). https://doi.org:10.1242/dev.119396
