# Supplementary material for: Uncovering developmental time and tempo using deep learning
Source: Nat Methods. 2023 Nov 23;20(12):2000–10. doi: 10.1038/s41592-023-02083-8 (PMC10703695; doi:10.1038/s41592-023-02083-8)
Supplement: Supplementary file 2 — Reporting Summary [file 41592_2023_2083_MOESM2_ESM.pdf]

nature portfolio

Corresponding author(s): Patrick Müller

Last updated by author(s): Oct 8, 2023

Reporting Summary

Nature Portfolio wishes to improve the reproducibility of the work that we publish. This form provides structure for consistency and transparency in reporting. For further information on Nature Portfolio policies, see our Editorial Policies and the Editorial Policy Checklist.

Statistics

For all statistical analyses, confirm that the following items are present in the figure legend, table legend, main text, or Methods section.

n/a	Confirmed	
☐	☒	The exact sample size (*n*) for each experimental group/condition, given as a discrete number and unit of measurement
☐	☒	A statement on whether measurements were taken from distinct samples or whether the same sample was measured repeatedly
☐	☒	The statistical test(s) used AND whether they are one- or two-sided<br>*Only common tests should be described solely by name; describe more complex techniques in the Methods section.*
☒	☐	A description of all covariates tested
☐	☒	A description of any assumptions or corrections, such as tests of normality and adjustment for multiple comparisons
☐	☒	A full description of the statistical parameters including central tendency (e.g. means) or other basic estimates (e.g. regression coefficient) AND variation (e.g. standard deviation) or associated estimates of uncertainty (e.g. confidence intervals)
☐	☒	For null hypothesis testing, the test statistic (e.g. *F*, *t*, *r*) with confidence intervals, effect sizes, degrees of freedom and *P* value noted<br>*Give P values as exact values whenever suitable.*
☒	☐	For Bayesian analysis, information on the choice of priors and Markov chain Monte Carlo settings
☒	☐	For hierarchical and complex designs, identification of the appropriate level for tests and full reporting of outcomes
☒	☐	Estimates of effect sizes (e.g. Cohen's *d*, Pearson's *r*), indicating how they were calculated

Our web collection on statistics for biologists contains articles on many of the points above.

Software and code

Policy information about availability of computer code

Data collection	For data acquisition on an Acquifer Imaging Machine we used the Imaging Machine control software (Acquifer Imaging GmbH, Version ID 4.00.21). Additionally, two Keyence BZ-X810 microscopes with the BZ-X800 viewer (Keyence, Version 01.03.00.01) were used. One of the Keyence BZ-X810 microscopes was equipped with a stage-top incubator (Oko-lab H301-KEYENCE-BZX with a UNO temperature controller).
Data analysis	For image annotation, we used the Visual Object Tagging Tool (Microsoft, https://github.com/microsoft/VoTT, Version 2.2.0). For model training and testing, we used custom Twin Network software (https://github.com/mueller-lab/TwinNet). The training was performed on NVIDIA RTX 3070 and 3090 cards (ASUS) on Windows 10 and Ubuntu 20.04, CUDA 11.2. For comparison to vector diffusion maps, we used software provided with the original publication (Dsilva et al., Development 2012) in MATLAB R2022a (MathWorks). Segmented time-series of single embryos were loaded into Fiji (ImageJ 1.54f), Adobe Illustrator (V. 26.2.1) and Adobe Photoshop (V. 23.3.1.426) for visualization and manual cropping.

For manuscripts utilizing custom algorithms or software that are central to the research but not yet described in published literature, software must be made available to editors and reviewers. We strongly encourage code deposition in a community repository (e.g. GitHub). See the Nature Portfolio guidelines for submitting code & software for further information.

Data

Policy information about availability of data

All manuscripts must include a data availability statement. This statement should provide the following information, where applicable:

- Accession codes, unique identifiers, or web links for publicly available datasets
- A description of any restrictions on data availability
- For clinical datasets or third party data, please ensure that the statement adheres to our policy

Training, evaluation and temperature data sets are available from https://doi.org/10.48606/50. Additional data used for training and evaluation are available from https://doi.org/10.48606/15, https://www.youtube.com/watch?v=M2ApXHhYbaw (accessed on 03/20/2023) and https://doi.org/10.7554/eLife.07410.021. Source data for all graphs is provided in separate source data files alongside this paper.

Human research participants

Policy information about studies involving human research participants and Sex and Gender in Research.

Reporting on sex and gender	N/A
Population characteristics	N/A
Recruitment	N/A
Ethics oversight	N/A

Note that full information on the approval of the study protocol must also be provided in the manuscript.

Field-specific reporting

Please select the one below that is the best fit for your research. If you are not sure, read the appropriate sections before making your selection.

☒ Life sciences ☐ Behavioural & social sciences ☐ Ecological, evolutionary & environmental sciences

For a reference copy of the document with all sections, see nature.com/documents/nr-reporting-summary-flat.pdf

Life sciences study design

All studies must disclose on these points even when the disclosure is negative.

Sample size	To determine a suitable sample size for the development of Twin Network as well as the segmentation network, we used an active learning approach. In an iterative process, we progressively increased the number of images/embryos used as training and validation sets until training metrics on the validation set reached a saturation level. The pool out of which the images were selected comprised more than 15,000 embryos. For data analysis sample sizes of at least 5 embryos were found to provide good accuracy (Fig. 4j and Extended Data Fig. 6f). Analyses of morphological variability and variability of predicted embryonic stages between normally developing embryos were performed on 77 embryos acquired in one experiment. Comparisons of morphological differences between normally and abnormally developing embryos were performed between 1 maldeveloping and 6 normally developing embryos from one image acquisition. Differences of predicted stages for normally and abnormally developing embryos were shown for 14 maldeveloping and 7 normally developing embryos from one experiment. Autostaging was performed on 131 zebrafish, 56 stickleback, 232 medaka and one C. elegans embryo. For the temperature analysis, 61 zebrafish and 146 medaka embryos were used for training. The zebrafish test data sample size per temperature was: n(23.5°C) = 211, n(25°C) = 198, n(26.5°C) = 209, n(28°C) = 168, n(28.5°C) = 126, n(30°C) = 187, n(30.5°C) = 102, n(31.5°C) = 130, n(33°C) = 98, n(34.5°C) = 70, n(35.5°C) = 119; and for medaka: n(18°C) = 65, n(21°C) = 32, n(23°C) = 92, n(26°C) = 47, n(28°C) = 46, n(30°C) = 41, n(31°C) = 21, n(32°C) = 40, n(33°C) = 42, n(36°C) = 35. For the comparison to the vector diffusion maps approach (Dsilva et al., Development 2012), 2 zebrafish embryos were analyzed.
Data exclusions	Embryos were excluded from data sets based on one of several criteria:<br>- Partial visibility in images<br>- Microscope image was taken without the embryo being in the correct focal plane<br>- Embryo showed visible signs of artificially induced or natural malformation during embryogenesis<br>- Particles obstructed the view of parts of the embryo<br>- Illumination of the embryo during acquisition was insufficient or unbalanced<br>- Embryos were unfertilized<br>- Tracking was inconsistent, e.g. due to extensive embryonic movement
Replication	Microscopy experiments for the provided training and testing data sets of Twin Network were carried out 34 times, and data was collected reliably and with comparable quality. Instructions for the replication of analyses using Twin Network and corresponding follow-along scripts are provided at https://github.com/mueller-lab/TwinNet. For embryonic age assessment experiments, at least three biological replicates were performed. The test data collection for the temperature analysis was performed once per temperature.

Randomization	Embryos from each species were randomly allocated into experimental groups.
Blinding	Since embryos from each species were indistinguishable in different experiments, blinding of the investigators was not necessary.

Reporting for specific materials, systems and methods

We require information from authors about some types of materials, experimental systems and methods used in many studies. Here, indicate whether each material, system or method listed is relevant to your study. If you are not sure if a list item applies to your research, read the appropriate section before selecting a response.

Materials & experimental systems

n/a	Involved in the study
☒	☐ Antibodies
☒	☐ Eukaryotic cell lines
☒	☐ Palaeontology and archaeology
☐	☒ Animals and other organisms
☒	☐ Clinical data
☒	☐ Dual use research of concern

Methods

n/a	Involved in the study
☒	☐ ChIP-seq
☒	☐ Flow cytometry
☒	☐ MRI-based neuroimaging

Animals and other research organisms

Policy information about studies involving animals; ARRIVE guidelines recommended for reporting animal research, and Sex and Gender in Research

Laboratory animals	We performed experiments exclusively on embryos and larvae that were not yet freely feeding. We used zebrafish of different genetic backgrounds to maximize the utility of the approach:<br>- Wild type TE (Pomreinke et al., eLife 2017)<br>- Tg(sebox:EGFP) (Poulain et al., Development 2002)<br>- Tg(gsc:GFP) (Doitsidou et al, Cell 2002)<br>- Tg(gsc:TurboRFP) (Sako et al., Cell Reports 2016)<br>- Tg(lhx1a:EGFP) (Swanhart et al., Int J Dev Biol 2010)<br>- sqt+/- (Dougan et al., Development 2003)<br>Age of embryos: 0-27 hpf.<br>In addition, medaka eggs of the Cab strain were used.
Wild animals	We did not use wild animals.
Reporting on sex	Sex-based analysis was not performed because phenotypical sex identification is not possible in early embryos.
Field-collected samples	We did not use field-collected samples.
Ethics oversight	All procedures involving animals were executed in accordance with the guidelines of the EU directive 2010/63/EU and the German Animal Welfare Act as approved by the local authorities represented by the Regierungspräsidium Tübingen and the Regierungspräsidium Freiburg. Experiments were executed exclusively on embryos and larvae that were not yet freely feeding.

Note that full information on the approval of the study protocol must also be provided in the manuscript.
